# Children’s perspectives on preventive youth health services: a focus group study among Dutch children

**DOI:** 10.1186/s12913-026-14824-4

**Published:** 2026-06-06

**Authors:** B. Bianca Fortuin, M. P. Mia Kösters, F. Francien Van Langen, J. M. M. Mai Chinapaw, M. H. H. Mariëtte Hoogsteder

**Affiliations:** 1 Public Health Service of Flevoland (GGD Flevoland), Department of Preventive Youth Health Care, Noorderwagenstraat 2, Lelystad, 8223 AM The Netherlands; 2 Amsterdam UMC, Location Vrije Universiteit Amsterdam, Public and Occupational Health, De Boelelaan 1117, Amsterdam, The Netherlands; 3 Amsterdam Public Health, Health Behaviors and Chronic Diseases, Methodology, and Quality of Care, Amsterdam, The Netherlands; 4 Public Health Service of Amsterdam (GGD Amsterdam), Department of Healthy Living, Nieuwe Achtergracht 100, Amsterdam, 1018 WT The Netherlands

**Keywords:** Public health, School health services, Youth, Participatory research

## Abstract

**Background:**

Children’s perspectives on optimal health care are rarely considered in health care research. These routine health assessments are carried out by Youth Health Care services (YHC), which provide school health care as part of preventive care for children. The aim of this study is to explore the perspectives of children aged 9 to 12 years old, on current routine health assessments in Dutch school health services and how these could be improved to better align their preferences and needs.

**Methods:**

We conducted a qualitative focus group study with Dutch primary school children, 9 to 12 years old, who received their routine health assessment from YHC in the past twelve months. Discussion topics included experiences with preventive youth health care services, preferences regarding involvement of parents, and suggestions for improvement of the services. Reflexive thematic analysis was conducted using the 6-step approach by Braun and Clarke.

**Results:**

Six focus groups were conducted with in total 41 children. We identified four main themes; ‘We want more! Monitoring health matters to children’; ‘Feeling safe and comfortable: be the nice and sweet professional in a two-way conversation’; ‘Parents know a lot, but not everything: mixed feelings on their role and presence’, and ‘Preventive youth health care should be organized more attractive’.

**Conclusion:**

Our sample of Dutch children perceived preventive routine health assessments as important to monitor their health and suggested more frequent and comprehensive assessments. The children’s perspectives, provided relevant insights in how to design child-friendly preventive youth health care.

**Supplementary Information:**

The online version contains supplementary material available at 10.1186/s12913-026-14824-4.

## Background

School health services exist in more than 100 countries worldwide, providing a wide range of services including vaccinations, screening and health promotion [1]. In the Netherlands, school health services are regulated under the national Public Health Act and delivered by preventive youth health care organisations, which provide public health care for all children from pregnancy and birth up to 18 years of age [2]. In primary school, routine health assessments are offered twice, at ages 5 and 10. These assessments include monitoring and screening for health and psychosocial problems, as well as support for parents and children and information on upbringing and healthy lifestyles. In addition, children are offered vaccinations against infectious diseases [2, 3].

School health services worldwide need to adapt to rapid societal changes, including digitalization, increasing mental health problems, chronic diseases, and widening health inequalities [1, 2, 4]. Organizational challenges such as staff shortages and inadequate financing further complicate this reformation [1, 2, 4]. Moreover, school health services play a key role in restoring the mental and physical health of children and adolescents following the COVID-19 pandemic [5]. These services can help children and adolescents catch up on preventive health care and maintenance, including vaccinations, hearing and vision screening and access to mental health support, all of which can have a positive impact on their development and academic performance.

Although preventive youth health care (YHC) can have a positive impact on children’s health [2, 6, 7], school health services are under increasing pressure, with less time available for each child due to limited funding and staffing [2, 4]. A study conducted in five European countries estimated that the spending on school health services is merely 0.16–0.69% percent of the total health care spending [8]. In addition, in the Netherlands, only four routine health assessments and vaccinations are offered to children in primary and secondary school, compared to 14 routine health assessments - including vaccinations, for children 0 to 4 years-old [9]. With these minimal resources, YHC is determining ways to reform health care efficiently and effectively, directed at the use of digital innovations and a triage approach, so that limited time and resources can be allocated more efficiently to children and parents who would benefit most [10–12].

To align health care services with children’s needs and preferences, it is important to engage them in the design and content of such services, as perspectives on health and health care may differ between children, professionals and parents [13–15]. An integrative review of school-aged children’s satisfaction with nursing care in acute care settings and a descriptive study from the United States comparing children’s and parents’ perspectives on nursing care, found that children considered it most important for nursing staff to be caring and helpful, whereas parents placed greater importance on staff competence [14, 15]. Regarding negative experiences, children in these studies identified painful procedures as the worst aspect of health care, whereas parents identified long waiting times as the worst aspect [14, 15]. Both children and adolescents were dissatisfied with how information about health issues and procedures was provided to them - and adolescents and health care providers differed in their perspectives on health care accessibility [13, 14]. Barriers for accessing healthcare varied on emphasis. On the one hand, adolescents mentioned lack of knowledge about the existence of services, difficulties with sharing health concerns and worries about negative judgements by health providers. On the other, health care providers mentioned practical issues such as opening hours or inadequate transport [13].

Since the introduction of the United Nations Convention on the Rights of the Child in 1989, children have been engaged more often in research about the development of interventions or health care innovations [16–18]. According to the Convention, children have the right to be heard and engaged in decisions that affect them [18]. In previous research, children expressed the wish to be specifically consulted, especially on issues affecting their life directly [19, 20]. From around age 7 years old, children are cognitive competent to participate in research and policy, such as the ability for logic thinking, judging their capabilities and expressing thoughts and feelings [21]. This enables them to make informed (medical) decisions and expressing their opinion on health, daily functioning, lifestyle and therefore being able to participate in research [22–24]. Although the Convention has been ratified by many countries in 1989, the perspectives of children and youth are still rarely engaged in studies on improving health care [25, 26]. Several studies investigated children’s and students’ perspectives on school health care, however, only one study engaged children 9 to 12 years old [27] while the other studies engaged younger children or adolescents older than 12 years [28–32]. Nursing care for children appears to be understudied, as, to our knowledge, the only review focused on satisfaction with nursing care in acute hospital settings among children aged 6 to 12 years [14]. Studies included in this review showed a low level of evidence, as most were qualitative in nature, with small samples and a wide age range, making it difficult to compare children’s experiences by age [14]. In addition, it is argued that nursing care in other contexts than the hospital, such as schools or primary care, is still a gap in research [14]. In order to learn more about children’s perspectives on satisfaction about routine health assessments in YHC, the aim of this study is to elicit the perspectives of children aged 9 to 12 years old, on current routine health assessments in Dutch YHC and how these could be improved to better align their preferences and needs.

## Methods

### Study design

We conducted a qualitative research study guided by a constructivist lens, featuring focus group methods among children aged 9 to 12 years old [33]. The constructivist paradigm assumes that knowledge is actively constructed by individuals through interactions with their environment, emphasizing the importance of understanding individuals’ subjective meanings and experiences [33]. Focus groups are rendered appropriate in cases where participants do not have a clear opinion on a subject matter and can therefore deliberate with peers on such matters [34]. In that sense, participants tend to develop their perceptions and opinions during the discussion [35]. Building on each other’s voice, a focus group facilitator stimulates and structures the groups’ thinking as well as inspires new ideas, and therefore might enrich the data [35, 36]. Furthermore, focus groups also create new ideas in a more natural way than in surveys or individual interviews [36]. Lastly, discussing topics surrounded by peers, reduces the researchers’ position of authority, which could be making the children feel more comfortable [21, 34].

### Participants and recruitment

Children were recruited from January 2024 to July 2025 through primary schools in Flevoland and Amsterdam-Amstelland, two regions in the Netherlands with approximately 100,000 primary school children, representing 7% of all children attending primary schools in the Netherlands [37, 38]. In these two regions, YHC organizations used different approaches for their routine health assessment. Both adhered to the YHC National Professional Framework, addressing health, upbringing, mental health, social development, and lifestyle [9]. In both regions the standard procedure was that all children were invited for a routine health assessment. Children were weighed and measured, and parents filled out a questionnaire. However, in Flevoland all children received a 20-minute consultation with the nurse, while in Amsterdam this will only occurred based on triage.

Children who had their routine health assessment from YHC in the past twelve months were eligible for participation. Purposive sampling was used to select schools with different educational philosophies in neighbourhoods with diverse characteristics, such as urban versus rural settings and socioeconomic position (based on welfare, the highest level of education attained by the main breadwinner and recent labour market participation) [39].

The researchers asked the YHC professionals, who were designated as contacts for specific schools, whether they would be willing to request for schools to participate in the study. If a school expressed interest, the YHC professional facilitated the connection between the researcher and the school. Schools that did not offer regular YHC services, predominantly special needs schools, were excluded. Of 32 schools that were approached, twelve were willing to participate. Reasons for non-participation included no time to participate, shortage of staff, or not interested. If an invited school agreed to participate, the researchers visited the school in person and informed the teachers and children about the study during school hours. The school board was informed by written information. Children who were interested to participate were provided an information- and consent letter, allowing them to decide, in consultation with their parents, whether to take part in the study. Six schools were excluded as less than six children showed interest or had parental informed consent.

### Procedures

When at least six children per school were willing to participate in focus groups, the sessions were held at the participating schools during school hours. Participants were seated in a circular arrangement to avoid placing moderators in a position of authority [21]. The sessions were conducted in Dutch which was the main language at the schools. All focus groups took 1 to 1.5 h and consisted of 5 to 6 children. The sixth focus group consisted of 14 children. This was larger than anticipated, and due to practical reasons, we were unable to split it into two smaller groups. All sessions were facilitated by the first author, who has a master’s degree health sciences and was trained in facilitating focus groups, assisted by a research intern, a final year master student in Medicine. The first author was a policy officer at GGD Flevoland, but not involved in conducting routine health assessments with children and therefore had no relationship with the participants.

Focus groups were conducted by two facilitators. The main facilitator led the session, while the co-facilitator assisted by keeping track of time, providing additional background information about the study and YHC and ensuring that all participants were seen and heard.

A discussion guide was developed by the research team (see Appendix 1.1). Questions covered topics such as children’s experiences with YHC services, specifically the routine health assessment, engagement of parents and children as well as suggestions for improvement.

The focus groups started with a playful and child-friendly introduction of both moderators and participants. Subsequently, information regarding the study, informed consent, and rules to create a safe environment were discussed. Children were asked if they would like to add rules, to limit a hierarchical relationship [40]. During the focus group, children were encouraged to freely discuss their thoughts, perspectives and ideas. Various kinds of energizers and engagement activities, such as short games, were used to promote fun and keep the children interested [41, 42]. There was a break halfway, if requested or deemed necessary by the moderators [21].

After every focus group, the moderators discussed their fieldwork observations and notes that could not be recorded and discussed their first impressions and remarkable observations [43]. After the first focus group, the researchers analysed the results, after which the questions were adjusted slightly [44].

Focus groups were conducted until the researchers determined that no new patterns of shared meanings were conceptualized, indicating that additional focus groups would not contribute further to the depth or richness of the data [40].

### Analysis

All focus groups were recorded and fully transcribed verbatim using the program cocreatewithai.eu [45]. Transcriptions were imported into MAXQDA qualitative software program (Version 24.5.1) which was used for managing and organizing the transcripts, as well as for coding and categorizing the data during the analysis process.

Reflexive thematic analysis was conducted, using the 6-step approach by Braun and Clarke [46, 47].

We used an inductive approach guided by a constructivism approach to structure the data, as code and theme development were directed by the subjective meanings and experiences of individuals within a specific context [33, 47]. The first step consisted of reading the transcripts, to check them with the recordings, getting familiarized with the data, starting to taking notes, and marking ideas for coding. During the second step of systematic data coding, two researchers (BF and FvL) highlighted and coded words or sentences which gave insight about children’s perspectives on the routine health assessments. The codes were interpreted by BF and FvL assisted by supervisors (MH and MK). We engaged in extensive dialogue about how to adequately represent the values of privacy, autonomy, and respect, which the children emphasized as important, and how these related to the roles of parents and professionals. It was a process of reading and re-reading of the transcripts, in consultation with and adding or refining codes. In the initial phase, we identified working themes such as ‘safe environment’, ‘being taken seriously’, ‘child-oriented’, and ‘importance of health’. After concluding that all codes reflected the dataset, initial themes were identified in step three, by examining the data for differences and commonalities, both with and across code categories, resulting in rearranging codes into groupings with shared meanings. Following an additional focus group and the further re-categorization and re-coding of the data, we observed a shift in how values of privacy, autonomy, and respect were positioned both between the child and the professional, and the child and the parent. We named the codes and themes, where appropriate based on words used by the children, to keep the data essence intact, reflecting participant’s meanings. In step four, initial themes were reviewed on the level of coded extracts and were identified in step five. During this phase, we had new discussions about the themes where we questioned whether two themes should be merged because they were very similar (‘Feeling safe and comfortable: be the nice and sweet professional, in a two-way conversation’ and ‘Preventive youth health care should be organised more attractively’ ). We identified different themes, as the first theme primarily focuses on the interaction between the child and the professional, while the other theme appears to have more of a practical/organizational focus. These discussions ultimately resulted in reworking some themes to capture the contours of the coded data. Subsequently, the transcripts were re-read and keyword searches were conducted to ensure that no relevant aspects of these themes were overlooked. We also ensured themes accurately reflected the data set and that they addressed the research question. Writing the report in the sixth step contributed to the final thematic structure, as successive draft versions and feedback from all contributing authors provided further insight into which themes and sub-themes best reflected the data set and addressed the research question.

### Ethical approval and informed consent

All procedures performed in studies involving human participants were in accordance with the ethical standards of the 1964 Helsinki declaration and its later amendments or comparable ethical standards. The Amsterdam UMC Medical Research Ethics Committee approved the study protocol (2021.0401 and A2021.0401.0001). All children provided verbal consent prior to participating in the focus groups, along with written consent from one parent, and, for children aged 12 years or older, their own written consent. Parents and children were fully informed about the study prior to the session, and consent was obtained separately from the verbal agreement. As an additional safeguard, all children were asked for verbal consent again before the focus group started, allowing them the opportunity to withdraw from participation if they no longer wished to take part. Data were transcribed and analysed anonymously. The audio recordings were only accessible by the researchers. The study was conducted and reported in accordance with the Reflexive Thematic Analysis Reporting Guidelines (RTARG) and COREQ guidelines (see Appendix A) where relevant and feasible [48, 49].

## Results

In total, six focus groups were conducted, in which 41 children were included (*N* = 41). Table 1 shows the main characteristics of all participating schools and children per focus group. Thematic analysis resulted in the generation of four themes; We want more! Monitoring health matters to children, Feeling safe and comfortable; be the nice and sweet professional in a two-way conversation, Parents know a lot, but not everything; mixed feelings on their role and presence, and Preventive youth health care should be organised more attractive. Although each theme highlights distinct aspects of children’s experiences of routine health assessments, these are not standalone elements but are interwoven to create a narrative of how children experience routine health assessments. While theme 1 focuses on the importance of routine health assessments, theme 2 and 3 examine how the interaction between children, YHC professionals and their parents influences children’s need for comfort, privacy and autonomy. Theme 4 finally provides insight into how YHC can address these needs from a more organizational and practical perspective.

**Table 1:** Characteristics participating schools and children for the focus groups

	Group 1 *N* = 5	Group 2 *N* = 5	Group 3 *N* = 6	Group 4 *N* = 5	Group 5 *N* = 6	Group 6 *N* = 14	Total *N* = 41
Gender, *n* (%)							
Boy	4 (80%)	2 (20%)	3 (50%)	4 (80%)	2 (33%)	7 (50%)	22 (54%)
Girl	1 (20%)	3 (40%)	3 (50%)	1 (20%)	4(66%)	7 (50%)	19 (46%)
Age, *M (SD)*	11 (0)	10.8 (0.45)	10.3 (0.82)	10.4 (0.55)	10.8 (0.45)	10.64 (0,63)	10.65 (0.57)
Characteristics school							
Urbanity	Suburban	Urban	Suburban	Urban	Suburban	Urban	Suburban = 3 Urban = 3
Denomination	Christian	Public	Christian	Roman Catholic	Christian	Christian	Christian = 4 Public = 1 Roman Catholic = 1
SEP *	High	High	High	Average	High	Low	High = 4 Average = 1 Low = 1
School size	> 200	< 100	170	100–200	> 200	100–200	< 100 = 1 100 – 200 = 3 >200 = 2

*socioeconomic position based on SES WOA score per ZIP-Code [38]

### We want more! Monitoring health matters

Children mentioned that it is important that their health is being monitored and that routine health assessments are useful and important for several reasons. First, children appreciated their health being monitored, and that YHC professionals register and keep track of their diseases. Second, children wanted to know whether they are in good health or not. Third, children saw the routine health assessment as an opportunity to detect, prevent and cure their diseases: “*And when you have something*,* you know how to get rid of it and how it was caused. Imagine*,* I have a disease caused by*,* let’s say something*,* by picking my nose too much. That is possible*,* but imagine that happens*,* then I learn I must not pick my nose too much.”* – participant 5, focus group FG 1.

Several children stated that the routine health assessment should be mandatory, free of charge, should take longer than the current appointment’s duration, and should be conducted more frequently, preferably every year. Children thought that five years between the current two assessments in primary school is too long, since someone’s health may change a lot over time: “*It could be that*,* imagine*,* you have a car accident or something*,* and then your health will change a lot. So*,* in five years*,* a lot can happen to your health.” –* participant 1, FG 2.

Children compared the routine health assessment with vaccinations, although these vaccinations were not offered during the assessment for 10-years old. Children seemed adamant that vaccination should not be a part of future assessments, because they disliked them. Children believed physical examination should be expanded beyond measuring height and weight, because they considered it important to detect health conditions, disorders, and abnormalities. Children specifically mentioned screening their eyes, ears, height, and weight. Other topics, such as bullying and feelings, were also mentioned as important to include in routine health assessments. Moreover, children expected better questions, advice, and education from the professional. Children thought YHC professionals should ask more targeted questions, to get a better insight into children’s health, such as ‘How are you feeling?’ or ‘Do you get bullied at school?’ , instead of ‘What sports do you do?’.

Children preferred advice about nutrition, their weight, or screen time, but the advice they received was perceived as too obvious and not specified enough to the child’s personal situation. Rather than telling children to ‘eat healthily’, YHC professionals should provide specific dietary advice to support healthier choices. Similarly, instead of simply advising increased physical activity, they should offer clear guidance on the types of sports or activities children could undertake: *“And then they say that you eat unhealthy. Uh*,* so what exactly should you eat then? What is unhealthy?”* – Participant 4, FG3.

Children expressed mixed views on whether puberty should be discussed during the routine health assessment at age 10, or that it would be more appropriate at older age. In one focus group, the importance of learning about puberty was highlighted, with particular emphasis on differences between boys and girls. One participant noted that understanding puberty is especially important for girls: *“Yes*,* especially for the girls.”* – Participant 2 and 3, FG2. Another participant added that boys might experience puberty later than girls and are therefore less likely to discuss it, but it is still important that they have the opportunity to do so if they wish: *“Well*,* probably. But I think boys experience it a bit later than girls*,* you know? So they don’t necessarily have to start talking about it*,* but if someone does bring it up*,* they can honestly admit whether they’ve experienced it or not.” -* Participant 1, FG2.

### Feeling safe and comfortable: be the nice and sweet professional, in a two-way conversation

Across all focus groups, children expressed a preference for a YHC professional who is kind and approachable during routine health assessments, in order to feel safe and comfortable: *“Just be sweet*,* have a smile on your face*,* talk kindly*,* talk more openly with each other.” –* Participant 3, FG2.

In one focus group, children reported encountering a YHC professional who was perceived as grumpy and serious, which made some children feel somewhat nervous. Children also indicated that they sometimes found it difficult to be open about their personal thoughts and feelings with someone they did not know. Some children did not mind talking to a stranger about their health, while others felt uncomfortable and express a desire to keep certain personal matters private; *“I didn’t really mind. I like getting to know people” –* Participant 1, FG 4. *“I just find it sometimes uncomfortable*,* and then I feel like… Yeah*,* I’m not going to say everything I do and what my mother doesn’t know about.” –* Participant 6, FG 5. Although some children appeared comfortable sharing personal information, they also believed that a YHC professional did not need to know everything: *“I dared to share things*,* but sometimes I just think that I have private things*,* and I just don’t want to tell them.”* – Participant 6, FG 5.

Although children preferred to speak with someone they already knew, they also acknowledged that this is not feasible for all routine health assessments. Therefore, they emphasised the importance of establishing a relationship of trust with the YHC professional by taking time to get to know one another. According to the children, this involves a two-way conversation between the child and the YHC professional. They also indicated that when the YHC professional shares some personal information, children find it easier to talk about themselves: “*If that person is also open before you open up. Anyway*,* for me*,* if that person is open*,* then I will think more like*,* I am going to be open.” –* participant 2, FG 2. Moreover, children believed YHC professionals should not rush during the routine health assessment. Instead, they should take the time to inform children about the purpose and content of the routine health assessment. More information in advance about the content and purpose would make the routine health assessment less tense, according to children: *“Maybe it could have been that she was going to do something you don’t know*,* and that you even don’t want to do that. […] She didn’t tell why. So*,* then you think*,* where*,* what are you going to do? Is it something scary*,* is something scary going to happen?” –* Participant 6, FG 3.

### Parents know a lot, but not everything; mixed feelings on their role and presence

Children’s views on the role and presence of parents during routine health assessments varied. Some children reported being open with their parents and considered it acceptable for them to be present during the assessment. They indicated a preference for parental presence, as parents possess substantial knowledge about the child. Some also considered parental presence beneficial, as it allowed parents to be informed immediately about any health concerns. In addition, parents could support the child during the assessment, reducing the need for the child to communicate everything independently: *“Sometimes it’s helpful for your parents to be there because they can answer questions that you might not really understand or notice.”* – Participant 11, FG 6. Children believed that from the age of 10, children could also talk to a professional on their own: *“…that from 10 or 12 years old you’re allowed to be alone*,* and that the parents then have to fill out a form saying they think you’re really ready to be alone now. And perhaps a short conversation afterwards with parents.”* – Participant 7, FG5. In another group a child suggested that: *“Maybe the parent could step out for a while*,* and then you can have a conversation with the nurse on your own.”* – Participant 4, FG 6. Moreover, when questionnaires need to be filled in, children said it is important that parents involve them as well. Another option mentioned was a separate questionnaire for children to fill in: “*The children should be helping with that because your parents do not know everything about you*,* you know it the best by yourself. So actually*,* being a bit more involved with the questionnaire…”* –Participant 3, FG3. Other children reported difficulty sharing personal information when their parents were present during routine health assessments; some because they did not usually discuss their problems or feelings with their parents and others irrespective of the nature of their relationship with them: “*Sometimes*,* I just want to say things that my parents don’t know. Sometimes my parents don’t know that I go to [supermarket] secretly.”* – Participant 5, FG4. Other children said they want to be taken seriously; they want their opinion to be heard. Therefore, they expressed it is annoying when parents interfere too much, doubt their perspectives, or correct their child afterwards: *“P2: My mother is also very annoying*,* […] she constantly says*,*'yeah*,* well that is your experience’. P4: Sometimes*,* my mother also says that*,* and I say*,* no but that is not my experience. P2: It is just true. P4: It is real.”* – Participants 2 and 4, FG1.

Children believed that it is important for both YHC professionals and their parents to respect their privacy. They indicated that they did not like their parents discussing topics they wished to keep private with a YHC professional or other family members. They felt that they had the right to decide whether to share certain information: “*It was okay that they talked with my mom. However*,* sometimes I thought; you do not have to tell them this or that.”* – participant 4, FG5.

### Preventive youth health care should be organised to be more attractive

Children described current routine health assessments as boring and sometimes uncomfortable. However, they also believed that these assessments are important and therefore do not necessarily need to be enjoyable. Nevertheless, they indicated that YHC organisations could make the experience more engaging. Children expressed a preference for more pleasant waiting and consultation rooms, for example by providing comic books and games: *“I had to wait half an hour. I could sustain*,* but a Donald Duck comic book or something else would have been nice.”* – participant 6, FG5. In addition, children recommended more activity during the routine health assessment such as, playing games, doing a sport, or filling in questionnaires or doing tests. Games could be health-related, but this was not necessary according to them. Games can be used during the introduction, to get used to each other, or as part of the actual assessment. To make a routine health assessment feel more comfortable, fidget toys could be used, or a pet could be present. Finally, some children preferred to be awarded with a small gift, as a sign of reward for doing their best during the routine health assessment. Children mentioned that they are used to this at other health care situations like the dentist or after drawing blood: *“P4: I would add something fun to do when you are being weighed. It is more fun when you have something fun to do. […] P5: A website*,* a questionnaire*,* I don’t know. Just something that it makes a bit*,* just something special*,* what makes you a little happy.”* – Participants 4 and 5 – FG2.

## Discussion

This study explored the perspectives of children aged 9 to 12 years on routine health assessments in school health services provided by Dutch YHC - and how these could be improved to better meet their needs. The findings suggest that children describe their experiences of routine health assessments through both practical expectations and relational meanings that influence their perspectives. Children considered these assessments important and believed they should occur more frequently. They also indicated that routine health assessments should give careful attention to their overall health, including more comprehensive physical checks and tailored advice on health and wellbeing. In their account, these expectations were closely tied to how they wanted to be approached. They emphasized the need for a child-centred approach; an environment in which their views are taken seriously, their privacy being respected and with clarity about what will happen. Children’s perspectives therefore portray the assessment not simply as a technical activity but as a context in which interpersonal engagement and professional conduct shape their sense of safety, comfort and engagement.

A notable finding is the contrast between children’s desire for more frequent and comprehensive routine health assessments and YHC’s current focus on optimizing efficiency. Organizations aim to allocate limited resources to children at risk, given challenges such as staff shortages and financial constraints [50, 51]. This tension highlights the complexity of balancing children’s preferences with organizational realities [1, 2, 50, 51]. Meanwhile, the cumulative impact of the COVID-19 pandemic, increasing digitalisation and the high prevalence of childhood overweight, mental health problems and chronic diseases underscores the urgent need to strengthen YHC services [1, 2, 4]. These challenges are compounded by widening health inequalities across Europe, driven by unequal access to healthy environments and care [2, 52–55]. The COVID-19 pandemic further exposed these disparities, reinforcing calls for stronger public health measures [55]. The absence of evidence and availability to age-appropriate screening tests and effectiveness of school-based intervention programs makes it hard to decide how to best allocate school health personnel [1, 2, 51]. Globally, regionally and nationally, there is a scarcity of data on the process of care, on how intended policies are implemented in practice and on the effects of YHC on children’s health outcomes [1, 2].

One potential solution to meet children’s needs for more frequent and comprehensive routine health assessments is the closer integration of health promotion within the educational system. The WHO has long advocated for schools as key settings for health promotion and education [56]. As every child has to attend school from an early age, schools could contribute to improving health and reducing inequality [57]. Moreover, schools play a vital role in the development of children’s social-emotional, cognitive and health-related competencies, which are salient for their ability to thrive as adults and contribute meaningfully to society [56, 58]. A growing number of governments acknowledge the strong connection between health, wellbeing and educational outcomes [56]. The widespread closure of schools during the COVID-19 pandemic has further highlighted this relationship [5]. The WHO defines a “Health Promoting School” as *“a school that constantly strengthens its capacity as a safe and healthy setting for living*,* learning*,* and working”* [5]. Such schools actively promote both education and the health and well-being of children. Topics related to health and well-being are integrated into the educational curriculum and can be taught by teachers, as well as by health care and social work professionals. Additionally, the preparation and provision of healthy meals may form part of this approach [56]. In many countries, Health Promoting Schools have been only partially implemented or not adopted at all [56, 58–60]. In the Netherlands, implementation remains limited - which is a missed opportunity, as preventive organizations and schools could substantially strengthen one another through collaboration [58, 60–62].

Children in this study also expressed a desire for additional screenings, such as vision and hearing tests. However, current guidelines restrict these examinations due to cost-effectiveness concerns [63]. For instance, Dutch guidelines indicate that vision screening beyond age seven is not supported for preventing amblyopia or refractive errors, as early detection occurs before this age and unnecessary screening may lead to overtreatment [64]. Explaining the rationale behind such decisions could help manage expectations and uphold children’s right to be informed [18]. Moreover, this would align with children’s expressed desires in multiple studies including our own to receive more information about the routine health assessment [27].

The quality of interaction between YHC-professionals, children and parents also matters. Children expected YHC professionals to engage with them through meaningful questions, advice and education delivered in a caring and patient manner, allowing sufficient time to actually get to know the child. This is consistent with studies about nursing care satisfaction, were ‘nurses as a person’ was the most highlighted category, emphasizing the importance of ‘humanity’ and positive personal characteristics -such as caring, being nice and smiling-, in nursing care across all child age groups [14, 65, 66].

The role of parents’ presence was a contested topic with varying perspectives. Previous literature identified parents’ presence as a factor influencing children’s engagement, at times inhibiting - and other times facilitating - their participation [66, 67]. In our study, some children valued their parents’ presence, while others felt it limited their ability to speak openly. Some children expressed a desire to speak with the YHC professional privately during the assessment, reflecting their need for autonomy and confidentiality as supported by the United Nations Convention on the Rights of the Child [18, 21]. However, Dutch legislation, specifically the Medical Treatment Agreement Act, requires parental consent for medical decisions and private consultations for children under 12 years of age [68]. This creates a tension between legal frameworks and children’s developmental rights [68]. Similarly, our findings reflect this complexity: children differed in their views on the appropriate ages for completing questionnaires independently or having private conversations with a professionals, suggesting a range between 10 and 12 years. Although research on shared decision making and children’s competence to provide informed consent is growing, practical questions remain regarding stakeholder roles and implementation [69, 70]. In the light of this consideration, we recommend offering children the option to interact with YHC professionals both with and without parental presence, balancing legal requirements with respect for children’s evolving rights and agency [18].

### Strengths and limitations

A strength of this study is the emphasis we placed on creating a safe and respectful space for children to share their perspectives during the focus group sessions, which we believe helped to enhance the authenticity and depth of the insights shared by the participants. This was confirmed by children mentioning that they felt safe and appreciated by both researchers’ attitude towards them. For example, they appreciated the sharing of personal information about ourselves at the beginning of the session - and the use of first names instead of formal titles like ‘teacher’ or ‘miss’. This contributed to an environment where they could openly share their perspectives and learn from each other. In addition, the children also said they liked our way of introducing ourselves and communicating with them. We observed that, once initiated, group discussions among the children evolved spontaneously into meaningful conversations. Children gave reasons for their opinions when different viewpoints arose, resulting in children learning from each other and sometimes changing their perspectives. We were unable to discuss and reflect on the final results of this study with the participating children. However, we plan to revisit these findings with a new group of children in a follow-up study, in which the results will be discussed and reviewed. A limitation of this study may be selection bias, as parents who did not want their child to participate in the routine health assessment may have been less likely to allow their child to participate in the study. During the focus groups, it was noted that 4 of the 41 participants had not attended the routine health assessment. We attempted to engage these children by briefly explaining the content of the routine health assessments and encouraged the other children to share their experiences. However, despite these efforts, it was challenging to engage these children in the discussion. However, this did not impact the overall results of the study. A strength of this study is its in-depth exploration of children’s perspectives on routine health assessments within two specific Dutch YHC regions. However, it is important to note that the findings may not be universally applicable to all children. Given the known regional and international variation in the organization and implementation of YHC services, the experiences of children in these regions may not reflect those of children in other regions. However, despite differences in organization between the two YHC regions included in this study, children from both regions expressed similar views on what they consider important in routine health assessments.

### Implications for YHC public health practice and further research

The findings of this study offer practical insights for enhancing preventive youth health care. Several recommendations can be implemented within existing procedures at minimal cost. For example, providing clear information to children before their routine health assessment, fostering a positive and trust-based relationship between the child and the professional and creating a more child-friendly environment, such as by providing a fidget toy or adding comic books in waiting rooms, are simple steps that can improve the child’s experience. These implications extend beyond YHC and can be used in every healthcare setting with children.

Beyond these immediate changes, the results underscore the importance of adapting routine health assessments to better reflect children’s perspectives. Children suggested various ways to make assessments more engaging and relevant, including interactive activities and personalized advice. However, implementing these ideas requires careful consideration of organizational constraints and professional standards. Future research ought to focus on developing innovative approaches that balance children’s preferences with the scope and quality requirements of YHC services. A participatory approach using a youth-led participatory action methodology may be particularly valuable for designing interventions that align with children’s needs while remaining feasible for health care providers. Such approaches ensure that children’s voices are not only heard but actively shape the development of preventive health care practices [71].

## Conclusion

This qualitative study provides valuable insights into how Dutch children, aged 9 to 12 years perceive routine health assessments within YHC. Despite the different challenges YHC is facing worldwide, our findings suggest that children favour more comprehensive, engaging and frequent routine health assessments. They emphasised the importance of building trust, respectful communication and clear information about the content, purpose and decisions related to routine health assessments. Children also highlighted the need for flexibility regarding parent engagement, reflecting children’s diverse preferences and evolving capacities.

These perspectives underscore the importance of designing child-centred preventive health services that balance efficiency with quality and respect for children’s rights. Incorporating children’s voices into service development can help to create assessments that are not only clinically effective but also meaningful and acceptable to those they are intended to serve.

## Supplementary Information

Below is the link to the electronic supplementary material.

Supplementary Material 1

## Data Availability

Data will be made available on request.

## Abbreviations

YHC: Preventive youth health care
FG: Focus group
