# Supplementary material for: Children’s perspectives on preventive youth health services: a focus group study among Dutch children
Source: BMC Health Serv Res. 2026 Jun 6;26:1110. doi: 10.1186/s12913-026-14824-4 (PMC13471172; doi:10.1186/s12913-026-14824-4)
Supplement: Supplementary file 1 [file 12913_2026_14824_MOESM1_ESM.docx]

1. Appendix

**Guide focusgroup 1**

| **Time** | **Description** | **Required materials** |
| --- | --- | --- |
|  | **Prepartion** |  Script   Recorder   Interactive whiteboard or laptop   PowerPoint presentation   Name tags   Pens |
| 10 min | **Sheet 1**  **Arrival:** Children enter and take their seats |  |
| 2 min | **Slide 2** Welcome, great to have you here! I’m Francien and this is Bianca. We are conducting research on the health check in grade 7, together with children from grade 7. We would like to know what you think about it.  First, we will introduce ourselves, then explain what we are going to do.  **Slide 3** I am Francien…  **Slide 4** I am Bianca…  **Slide 5** What will we do today? Today, we want to hear from you what you think about the health check you had in grade 7. To do this, we will first explain some things and make agreements together. During the conversation, we will play some games and, for example, do some drawing. Meanwhile, we will ask questions and have a discussion together. The session will last a little over an hour in total. |  |
| 1 min | **Slide 6: Explaining the rules** Before we continue, I would like to come up with a few rules together with you. I have already thought of the following rules:   - You don’t have to agree with each other. You don’t have to convince others. We want to hear different opinions. Of course, you can change your opinion if you hear what others think. There are no right or wrong answers; we don’t have the answers. This is not a test. We are researchers ourselves and look critically at the health check. We did not create or design it, nor do we carry it out. If you say something negative about the health check, you cannot hurt us with that. We actually want to know. - You don’t have to answer a question if you don’t want to. You don’t have to say anything about your own health. You can share it as an example if you want, but today it’s about what you think of the health check. - Say if I don’t understand you or if you don’t understand me. - Speak one at a time. - Everything shared here stays here. Don’t make fun of others or joke about them, not even after the group. - And we think it’s important that you have a fun session and enjoy yourselves!   Do you have any other rules or agreements we should add? Are there any questions before we start?  **Slide 7: Mentioning audio recordings** We will record everything during the conversation. We will also occasionally check if the microphone is still on. We will transcribe the audio recordings. When we transcribe the recordings, we make sure your names are not included. This way, it stays anonymous. Do you know what anonymous means? After the recordings have been transcribed, we will delete the audio files. The transcribed file will be stored in a secure place. That’s why it’s especially important not to talk over each other, because the microphone doesn’t record that well. |  |
| 3 min | **Slide 8: Warm-up game/Energizer** Tick Tack Boom Throw a ball and take turns asking each other a question and answering it. If you don’t want to answer a question, you have to do a spin. With a timer—when it goes off while you’re holding the ball, you’ve ‘lost’**.** | Ball or tick tack boom game  Timer (phone) |
| 5 min | **Slide 9** Question: What do you think of when you hear GGD/Youth Health Care? Or as a question Or as a game with Tick Tack Boom |  |
| 1 min | **Slide 10: Explaining the topic** Youth Health Care monitors the health of children, checks whether you are growing well, and whether you are all happy. You also receive vaccinations from Youth Health Care, but we won’t be discussing those today! Youth Health Care conducts health assessments. For babies, this health assessment takes place at the well-baby clinic. Hopefully, this year you have also had a health assessment with a nurse, which might have been called the school doctor? They weighed and measured you and had a conversation with you. We would like to know what you thought of this health assessment. Is it clear to everyone now what the health assessment is? For the rest of this conversation, we will call it the “health assessment”—that’s what we mean by it. Do you have any questions? |  |
| 5 min writing or drawing  5 min plenary discussion | **Slides 11 & 12, skip if already answered**  Can you remember having the health assessment? Did you all have a health assessment?  Or question: What exactly did your health assessment look like?  Or task: News bulletin: Write a report for the (school) newspaper about what exactly happened during the health assessment. You can also do this by drawing a picture or making a comic strip. Make sure to answer as many of the following questions as possible:   - How did you hear about the health assessment? - How did you decide to participate? - What happened during your health assessment? - Where did the health assessment take place? - Who was with you? - Would you do it again?   Questions: What did you write down? Are there any differences or similarities? | Paper  Pens  Pencils  Markers |
| 10 min  1 min | **Slide 13** Round-robin question, so everyone answers the question in turn.  Would you recommend participating in the health assessment to other children? Why or why not?  **Dia 14: Then a brief explanation of why the health assessment is conducted.**  All children in the Netherlands have the right to grow up as safely and healthily as possible. Youth Health Care monitors the health and safety of every child from the moment the mother is pregnant until you turn 18. They do this by carefully checking growth, health, development, and safety at various moments. If there is a chance you are not growing well or are at risk of developing an illness, you can be treated in time before you actually become ill. This can include extra conversations about healthy eating or the nurse arranging an appointment at the hospital. Youth Health Care also tries to detect bullying and child abuse through the health assessment. |  |
| 3 min free listing  15 min plenary discussion | **Slide 15: Task** Free listing: Write down as many things as possible about the following two questions, all together on a large sheet of paper. Make a sort of pros and cons list. You can also make drawings. You have 3 minutes.   - What was helpful to you? - What was not helpful to you?   **Slide 16** Look at the poster.  What would make the assessment more useful? When do you benefit from the assessment? What is the right time for the health assessment? Can you tell us what stands out to you? Who wrote this down? Please tell us more about it. Is there anything missing that hasn’t been written down yet? When you look at the poster, what is your conclusion? | Large posters Post-its  A4 sheets  Pens, colored pencils, markers |
| 15 min | **Slide 17 Statements:**  Either stand in a line to show how much you agree OR hold up green and red cards  **Slide 18** I was talked to, or people talked about me. Follow-up question: What do you think about that?  **Slide 19** I dared to ask/say everything. Follow-up questions:   - How was it to talk with a complete stranger? - Did you already know the nurse? - How did you feel about your parents being there? - Did having your parents there make you ask more or fewer questions? | Green and red pieces of paper |
| 10 min | **Slide 20 Finishing sentence:** Thinking about everything we discussed today, complete the following sentence:  If I were in charge, I would change the health assessment by... And what would you keep the same? If you had a magic wand and anything was possible, how would you design the health assessment? |  |
| 5 min | **Slide 21** Brief evaluation Of the focus group:  What did you like about the focus group and what didn’t you like? What did you find clear about the questions and what didn’t you find clear? Do you have any ideas about what we could do better or differently in the next session? |  |
|  | **Slide 22: Closing** Distribute cards/Post-its.  Final question: Write down anything else you want to share, something you maybe didn’t dare to say, or anything that just comes to mind about the topic on a card/Post-it. When you’re done, you may go back to class. | Cards/post-its |

**Checklist / Questions to be answered:**

- What do the children want to change about the health assessment? (Slide 16 & Slide 20)
- What do the children find useful about the health assessment? (Slide 15)
- What do the children find not useful about the health assessment? (Slide 15)
- Do the children feel that they are talked to or talked about? (Slide 18)
- Do they feel involved in the health assessment? (Slide 18)
- Do they think that involvement should be different? (Slide 18)
- How do they feel about talking one-on-one with a nurse? (Slide 19)
- Do they feel comfortable with a nurse they don’t know? (Slide 19)

**Overview of questions:**

1. When I think of the GGD, I think of: (Slide 9)
2. Do you remember having the health assessment? How exactly did it go? (Slide 11)
3. Would you recommend other children to participate in the health assessment? (Slide 13)
4. Free listing: (Slide 15)
   a. What was helpful to you?
   b. What was not helpful to you?
   c. What would make the assessment more useful? (Slide 16)
   d. When do you benefit from the health assessment?
   e. What is the right time for the health assessment?
5. Statements:
   a. I was talked to, or people talked about me. (Slide 18)
   b. I dared to say or ask everything. (Slide 19)
   i. How was it to talk to a complete stranger?
   ii. How did you feel about your parents being there or not?
6. Finish the sentence: If I were in charge of the GGD, I would change the health assessment by… (Slide 20)
7. Evaluation: What did you think of it? Any highlights or tips? (Slide 21)
8. Closing: Write down anything else you want to share on a card/Post-it. (Slide 22)

**Topics:**

- Involvement of parents/children
- Process of the health assessment
- Setup of the health assessment
- Timing of the health assessment
- Before and after the health assessment

**General schedule:**

- 20 min arrival/introduction
- 30 min warm-up questions (part of data collection)
- 25 min key questions
- 10 min closing/evaluation
